# Chemosensitization strategies for the treatment of lung cancer

**DOI:** 10.18632/oncoscience.208

**Published:** 2015-08-20

**Authors:** Laura Senovilla, Guido Kroemer

**Affiliations:** Université Paris Descartes, Sorbonne Paris Cité; Equipe 11 labellisée Ligue Nationale contre le Cancer, Centre de Recherche des Cordeliers; Institut National de la Santé et de la Recherche Médicale, Université Pierre et Marie Curie, Metabolomics and Cell Biology Platforms, Gustave Roussy Cancer Campus; Pôle de Biologie, Hôpital Européen Georges Pompidou, AP-HP; Paris, France

**Keywords:** non-small cell lung cancer, breast cancer, meta-analysis of microarrays, melanoma, colorectal carcinoma

In spite of major research efforts, the treatment of advanced non-small cell lung cancer is still largely inefficient. Indeed, this cancer type has a poor prognosis, and no curative therapies are available.

Over the last years, it has been become increasingly clear that (relatively) successful chemotherapies such as the anthracycline-based neoadjuvant treatment of locally advanced breast cancer are largely influenced by the immunosurveillance system. Thus, the pre-treatment composition of the immune infiltrate of mammary carcinomas determined by microarray analyses clearly affects the probability of successful therapy. A CXCL13-centered metagene signature reflecting the intratumoral presence of interferon-γ-producing T cells has a positive predictive impact, indicating that the pre-existing anticancer immune response influences therapeutic outcome [1]. In addition, changes in the immune infiltrate induced by chemotherapy have a prognostic impact. Complete pathological responses observed after six cycles of anthracycline-based chemotherapy are associated with an improvement of the ratio between CD8^+^ cytotoxic T lymphocytes (CTL) and immunosuppressive FOXP3^+^ regulatory T cells [2]. Thus, it appears that chemotherapy can indeed elicit anticancer immune responses.

One important mechanism through which chemotherapy stimulates anticancer immunosurveillance consists in the induction of immunogenic cell death (ICD)[3]. ICD is a cell death modality that is preceded by cellular stress responses (in particular autophagy, endoplasmic reticulum stress, as well a type 1 interferon production) that affect the perception of dying cells and their corpses by the immune system. *Premortem* autophagy is required for the optimal release of ATP, and extracellular ATP is (one of) the major chemotactic factor(s) that attracts myeloid cells into the proximity of dying cancer cells. Endoplasmic reticulum (ER) stress facilitates the exposure of ER luminal proteins such as calreticulin on the surface of the plasma membrane, and surface-exposed calreticulin serves as a potent ‘eat-me’ signal facilitating the transfer of dead-cell antigens into immature dendritic cells. HMGB1, which leaks out from the nuclei of dead cells, stimulates the maturation of immature dendritic cells, which then can present tumor-associated antigens to CTL. Type 1 interferon is required for conditioning the tumor microenvironment to optimize the recruitment and action of CTL [3-5].

It is important to note that anthracyclines and oxaliplatin are efficient ICD inducers, perhaps explaining that these drugs can be successfully used for the adjuvant or neoadjuvant treatment of mammary and colorectal carcinomas, respectively. In contrast, cisplatin, which is widely used as the first-line treatment of lung cancer, is a relatively poor ICD inducer, presumably because it fails to stimulate an efficient ER stress response [3].

**Figure 1:**
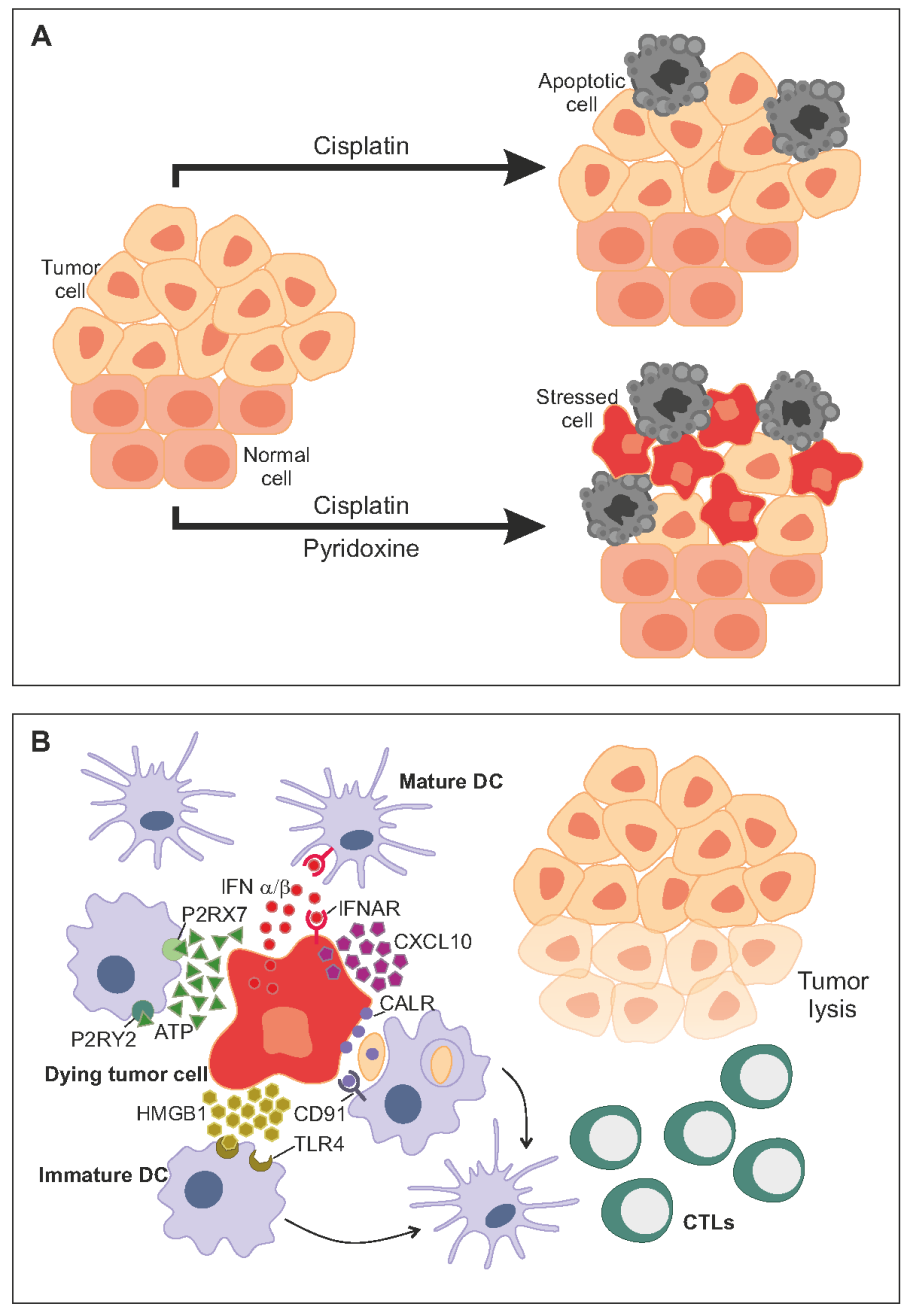
An optimal strategy for chemosensitization **A.** Quantitative goal. Two drugs should kill more cancer cells when combined among each other than when they are used separately. **B.** Qualitative goal. Two drugs should induce all features of immunogenic cell death (ICD) when they are combined.

Many non-small cell lung cancers (NSCLC) are primarily resistant against cisplatin, a feature that can be explained by their metabolic characteristics. Thus, the levels of expression of pyridoxine kinase (PDXK) by NSCLC cells have a major prognostic impact on the survival of patients treated with cisplatin[6]. PDXK is the enzyme that converts cell-permeable pyridoxine (also called vitamin B6) into pyridoxine phosphate, the active metabolite that is trapped in cells and can serve as prosthetic group for multiple enzymes. Pyridoxine sensitizes NSCLC cells to the induction of apoptosis by cisplatin, but only if PDXK is expressed, meaning that it is indeed the intracellular level of pyridoxine phosphate that modulates the cisplatin response [6]. Importantly, pyridoxine does not only shift the dose response to cisplatin to lower levels with regard to apoptosis. Pyridoxine also enhances the efficacy of cisplatin with regard to the induction of the ER stress response, thereby improving the potential of the drug to trigger ICD (Figure 1). As a result, cisplatin and pyridoxine can be advantageously combined for the treatment of mice with lung cancers. The synergistic interaction between cisplatin and pyridoxine is largely dependent on an adaptive anticancer immune response. Cisplatin plus pyridoxine can cure immunocompetent mice bearing orthotopic lung cancers, yet fail to achieve complete responses in nude mice, which lack T lymphocytes [7, 8]. Moreover, mice that have been cured from NSCLC by the combination therapy develop an effective immune response, making them resistant against re-challenge with NSCLC cells.

Altogether, these findings support the notion that optimal chemosensitization strategies should pursue two parallel goals, namely (i) to render the cancer cells more susceptible to lethal responses and (ii) to seek maximum efficacy in the induction of ICD (Figure 1). In other words, ICD should be routinely monitored for the development of novel combination therapies. Only those combinations that facilitate optimal stimulation of ICD and are compatible with the induction of an anticancer immune response will be clinically successful.
